# Extragonadal Pelvic Yolk Sac Tumor in a Postpubertal Patient: Case Report With Radiologic Correlation and Review of the Literature

**DOI:** 10.1002/ccr3.70939

**Published:** 2025-09-24

**Authors:** Saurav Jha, Emilee Phillips, Martin Jumbelic Safran, Saurabh Gupta

**Affiliations:** ^1^ Department of Radiology Patan Academy of Health Sciences Kathmandu Nepal; ^2^ Department of Radiology SUNY Upstate Medical University Hospital New York USA; ^3^ Department of Pathology SUNY Upstate Medical University Hospital New York USA

**Keywords:** case report, lump, pelvis, ultrasonography, yolk sac tumor

## Abstract

Yolk sac tumor, also referred to as endodermal sinus tumor, is an aggressive malignant germ cell neoplasm that most commonly originates in the gonad. Its occurrence in extra‐gonadal sites is infrequent and is highly malignant, with only a handful of cases reported in the literature. With the help of this article, we want to make readers aware of the rare site of yolk sac tumor, its clinical presentation, and its radiological findings. As clinicians, we should be vigilant to consider differential diagnoses for an underlying etiology, like yolk sac tumor, for a patient presenting with a subcutaneous lump. Even though swelling in subcutaneous regions, in most cases, represents a benign diagnosis, malignant ones should always be kept in the back of the mind while managing patients. Variation in the site of extragonadal germ cell tumors has been highlighted with this paper, and herein, we aim to make readers aware of a rare and uncommon germ cell tumor, acknowledging the importance of imaging findings in making a diagnosis and the approach for its management.

## Graphical abstract

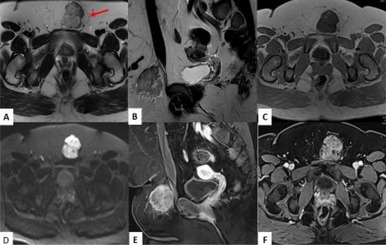

## 1 Introduction

Yolk sac tumor (endodermal sinus tumor) represents one of the rare germ cell neoplasms arising from the gonads. Among all the germ cell neoplasms, 90% of them arise from the gonads and only 10% represent extra‐gonadal origin, with common sites of extra‐gonadal location being the sacrococcygeal area, mediastinum, central nervous system, liver, omentum, and pineal gland [1]. Its location in the pelvis is relatively rare, and to the author's knowledge, this is the first reported case of a pelvic extra‐gonadal germ cell tumor with presentation as a subcutaneous lump.

Among mixed germ cell tumors of the ovary, the yolk sac tumor represents 33% of cases in the premenarchal age group, with the usual age group of presentation varying between 18 and 35 years of age [2]. These tumors are among the few highly malignant neoplasms that fall under germ cell tumors and have a high response to chemotherapy. Clinical manifestation is highly variable, with features depending upon the site of location, and the diagnosis is usually made with a combination of history, clinical examination, imaging findings, tumor markers, and histopathological pictures. USG features include a heterogeneous mass with both echogenic and hypoechoic areas, while MRI features include an isointense lesion in the T1‐weighted image and hyperintense in the T2‐weighted image. Other than the imaging finding, AFP also serves as an important tumor marker in diagnosing and monitoring the response to treatment [3]. Imagine finding suspicion of the yolk sac tumor is confirmed with the histopathological features and tumor markers stain for the neoplastic cells.

This case report highlights a rare site of extra‐gonadal germ cell tumor and its clinical features, along with its radiological characteristics. Our aim is to highlight how a seemingly innocuous subcutaneous lump can be a sign of an extremely rare site of a rare malignancy and the importance of keeping it as a differential diagnosis for an abdominal lump. It provides insight into the dynamics of the extra‐gonadal germ cell tumor and highlights the importance of the radiological finding in guiding the diagnosis and management.

## 2 Case History/Examination

A 19‐year‐old female was referred to our hospital with chief complaints of a tender mass in the hypogastric region for 1 year, with an increase in size 2 months prior to hospital admission. On further enquiry, it was noted that the patient had a history of appendicitis for which she underwent a laparoscopic appendectomy 3 months back with a normal course during the hospital stay. There was no history of trauma in the area, prior bruising, drainage, or any other problems during the hospital stay. The patient did not have a history of fever, night sweats, weight loss, redness, warmth, discharge, or bowel and bladder dysfunction.

On examination, a lump was located in the hypogastric region near the pubic symphysis area with a maximum length of 4 cm and a breadth of 3 cm. It had a smooth surface, with an ill‐defined margin, hard consistency, and no local rise in temperature. The mass was non‐compressible with no pulsatility, semi‐fixed to the underlying structures, and had negative transillumination with no regional lymphadenopathy. All the lab investigations were normal except for raised tumor markers like AFP and CA125, which subsequently decreased with the treatment.

## 3 Differential Diagnosis, Investigations, and Treatment

**FIGURE 1:**
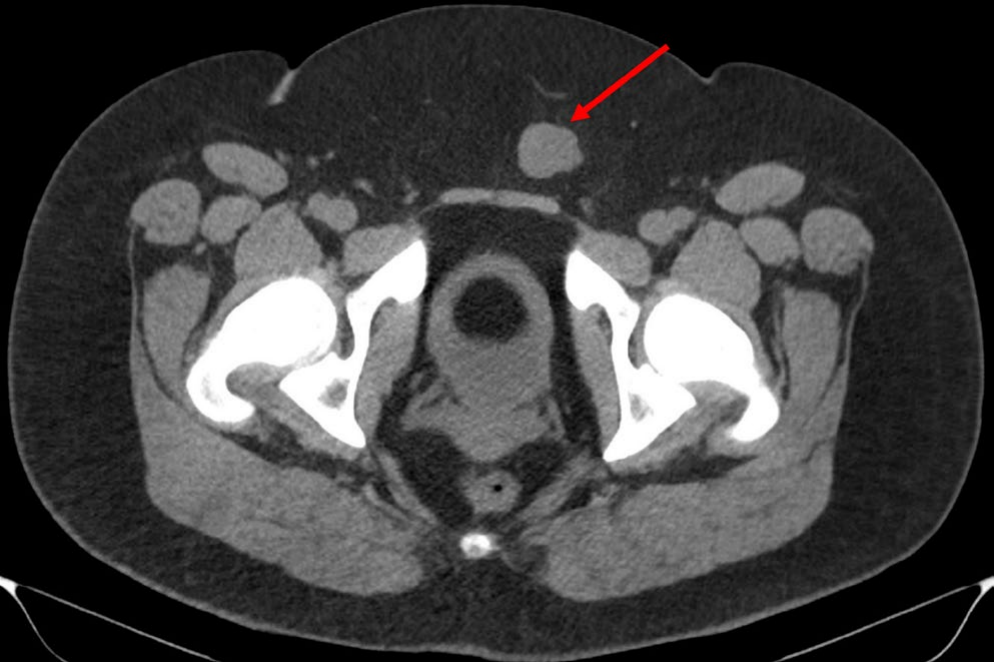
CT abdomen and pelvis without IV contrast performed 5 months prior to the patient's diagnosis reveals a round mass in the subcutaneous tissues anterior to the pubic symphysis that is isodense to muscle (red arrow). At this time, the mass measured 2.0 × 2.7 cm (AP × TR) with no significant surrounding edema.

**FIGURE 2:**
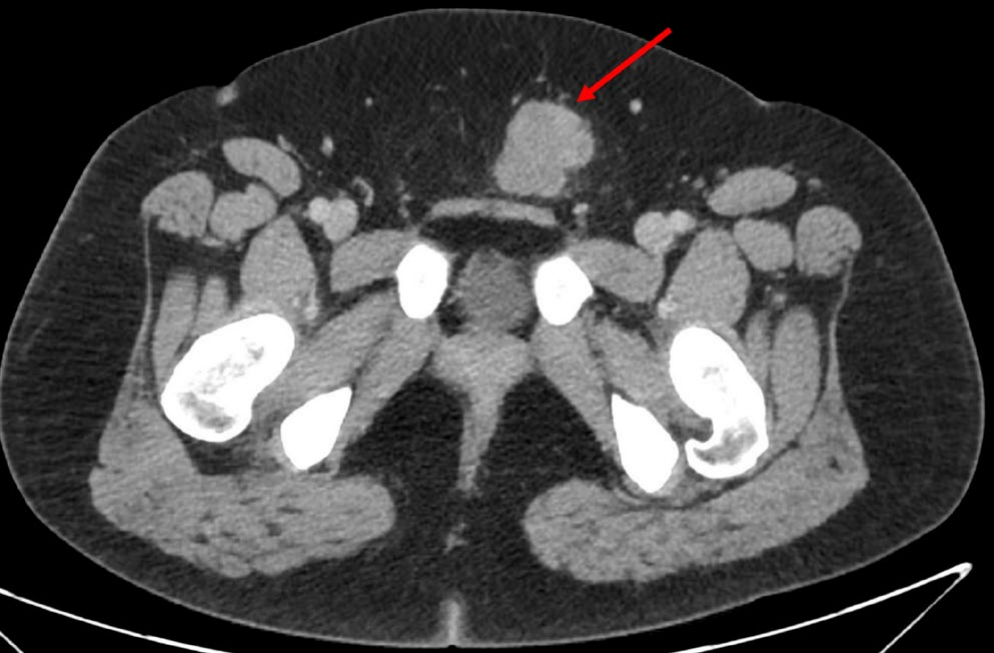
Follow‐up CT abdomen and pelvis with IV contrast performed approximately 1 month prior to the patient's diagnosis. There is interval enlargement of the anterior abdominal wall mass now measuring 3.2 × 2.8 cm with a minimal amount of surrounding fat stranding (red arrow), with the mass showing increased lobulation of the margins. This was thought at the time to represent either a solid mass or hematoma, and ultrasound was recommended to evaluate the vascularity of the lesion.

**FIGURE 3:**
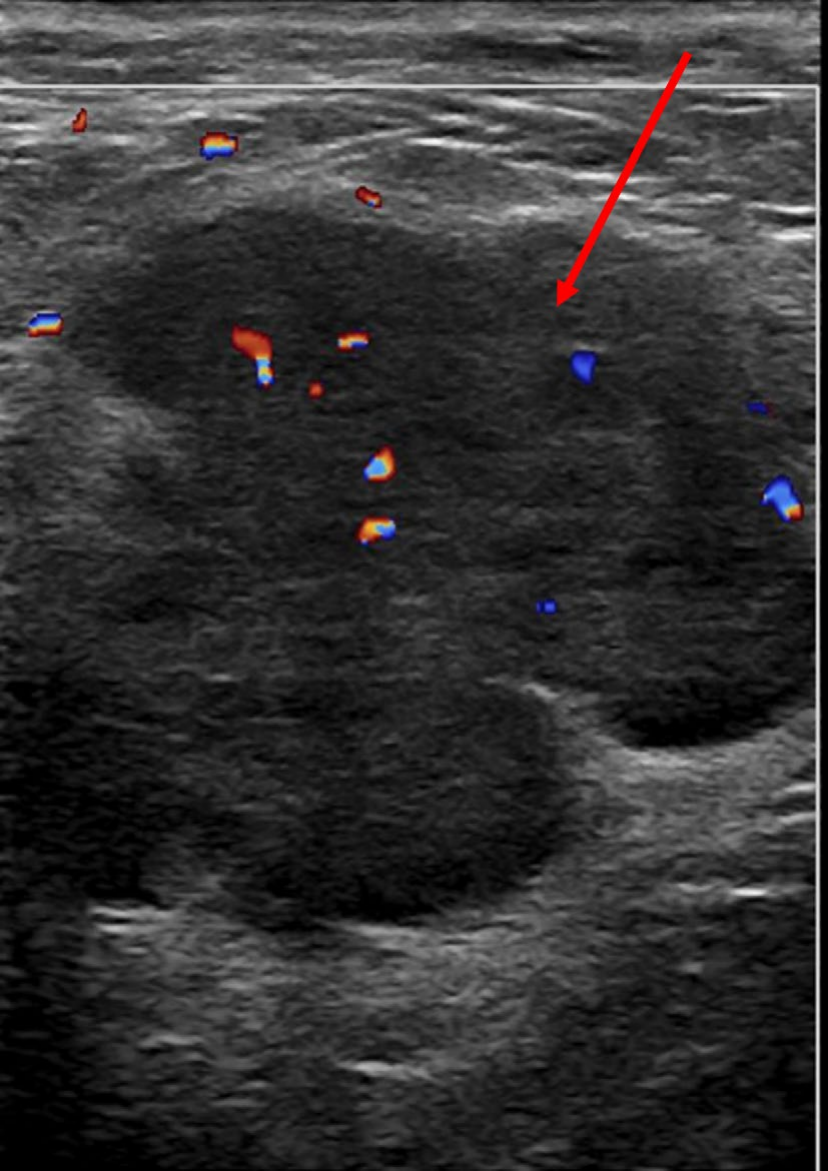
Transabdominal ultrasound of the suprapubic region performed the same day as the CECT. The image demonstrates a hypoechoic irregular mass with lobulated borders, internal vascularity on color doppler (red arrow), and the lesion measured 4.7 × 4.2 × 3.2 cm.

**FIGURE 4:**
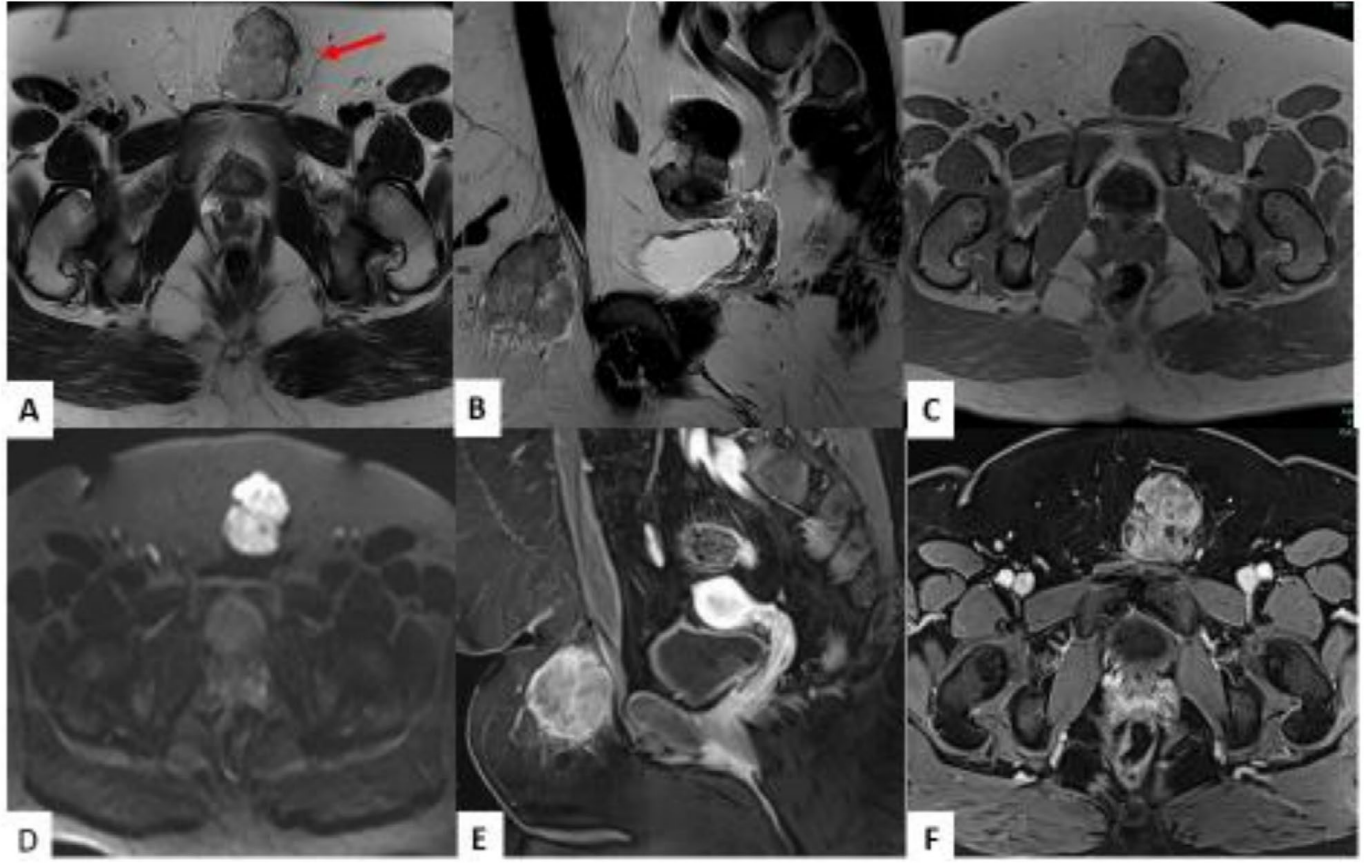
MRI of the pelvis with and without contrast performed 3 days prior to biopsy. Axial (A) and sagittal (B) T2‐weighted MRI of the anterior abdominal mass demonstrating heterogeneous T2 hyperintensity. Axial T1 (C) weighted image of the mass illustrates its relative T1 hypointensity. Axial diffusion weighted image (D) of the mass shows avid diffusion restriction. Corresponding ADC mapping (not shown) is hypointense in the areas of DWI hyperintensity. Sagittal (E) and axial (F) post‐contrast T1‐weighted images demonstrate heterogeneous enhancement of the mass.

**FIGURE 5:**
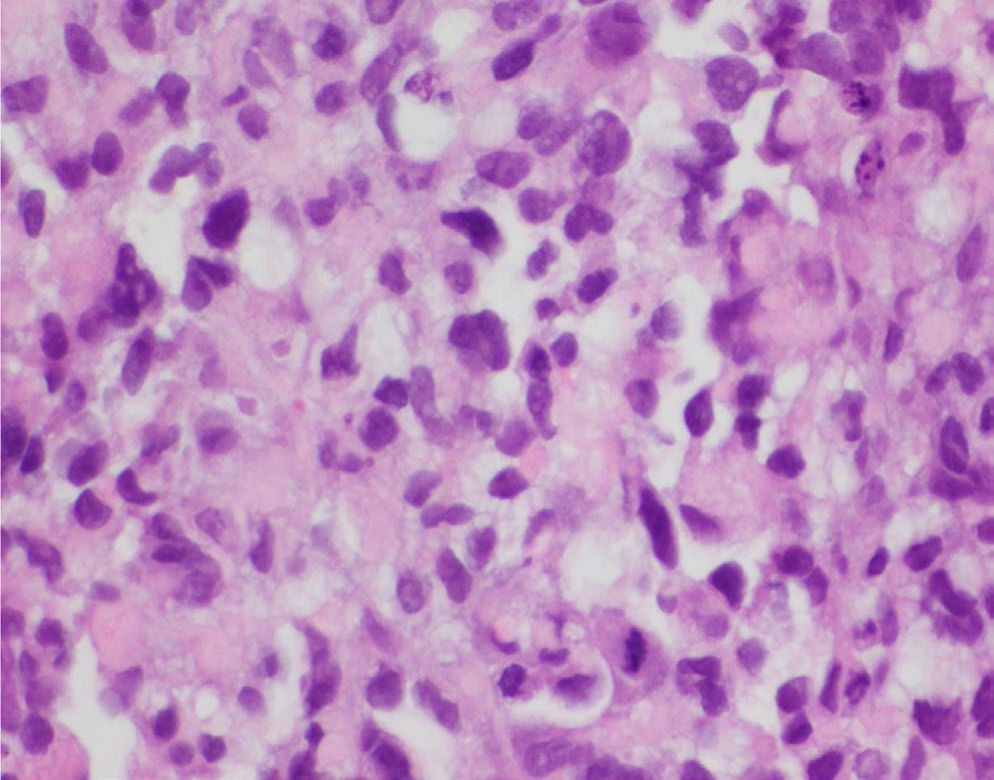
Histological evaluation of the specimen revealed abundant eosinophilic cytoplasm, round nuclei, and variable mitotic activity (hematoxylin and eosin stain ×40).

**FIGURE 6:**
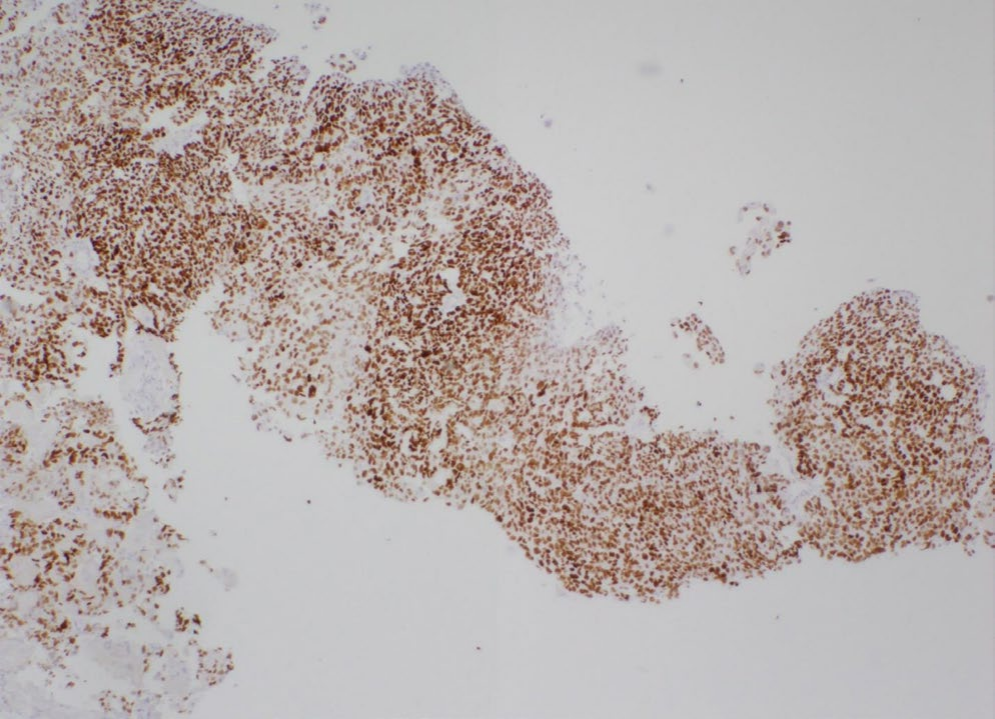
Immunohistochemical staining shows that neoplastic cells are positive for SALL4.

**FIGURE 7:**
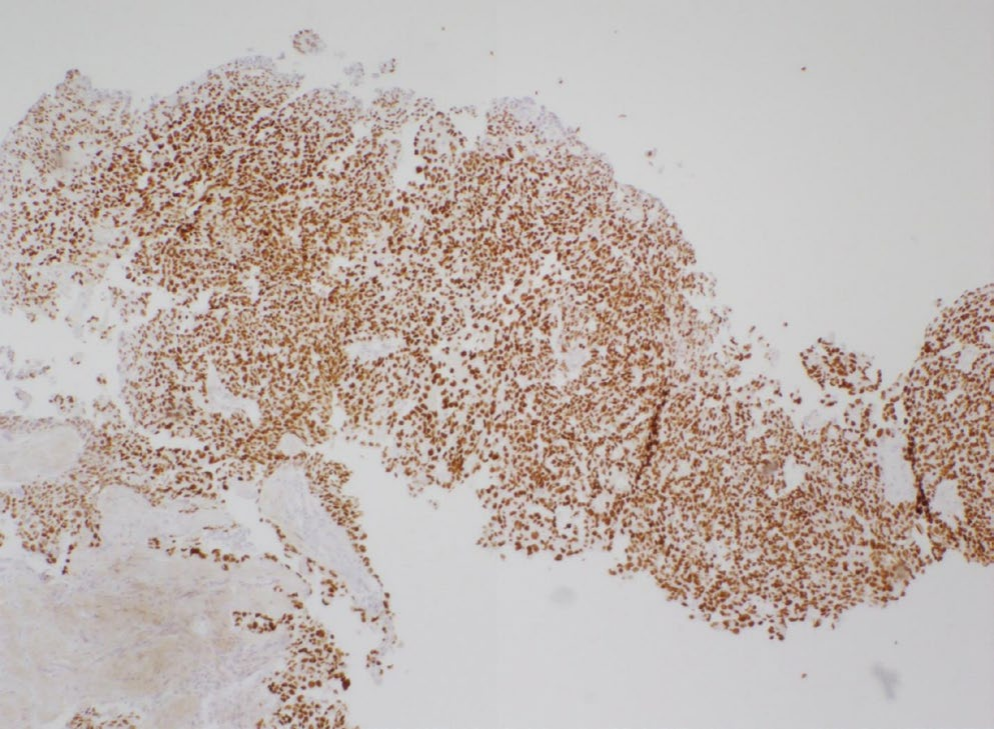
Immunohistochemical staining shows that neoplastic cells are positive for CDX2.

**FIGURE 8:**
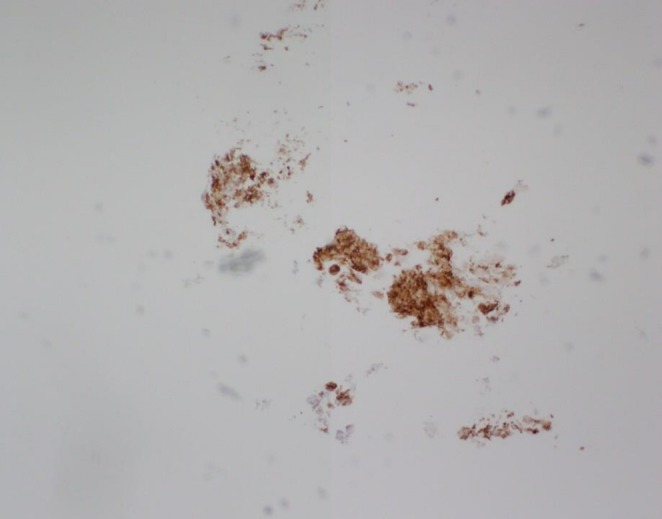
Immunohistochemical staining shows that neoplastic cells are positive for glypican 3.

**FIGURE 9:**
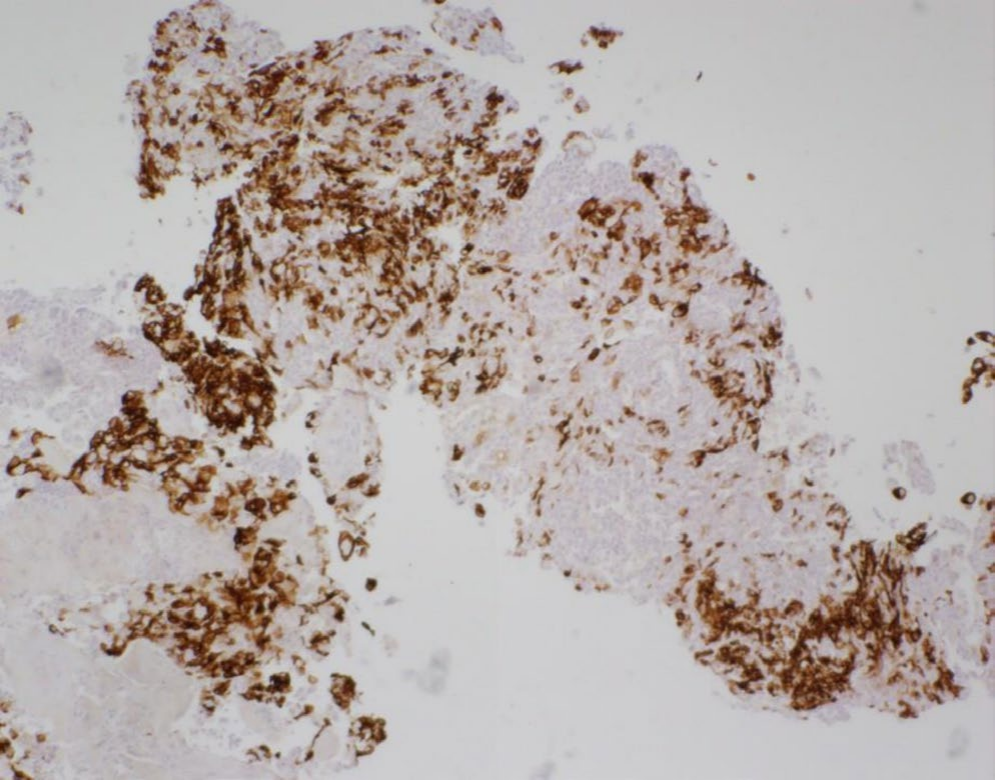
Immunohistochemical staining shows that neoplastic cells are positive for cytokeratin 7.

Plain CT abdomen and pelvis performed 5 months prior to the diagnosis revealed a round mass in the subcutaneous tissue anterior to the pubic symphysis that was isodense to muscle and measured 2.0 × 2.7 cm with no surrounding edema (Figure 1). Follow‐up CECT abdomen and pelvis with IV contrast performed 1 month prior to the diagnosis revealed interval enlargement of the mass measuring 3.2 × 2.8 cm with a minimal amount of fat stranding along with increased lobulation of the margin (Figure 2). Transabdominal ultrasound of the suprapubic region was also done on the same day of CECT and revealed an irregular hypoechoic mass with a lobulated border and internal vascularity on color Doppler, measuring 4.7*4.2*3.2 cm (Figure 3). MRI of the pelvis with and without contrast performed 3 days prior to biopsy revealed a hyperintense anterior abdominal mass on the T2‐weighted sequence in both axial and sagittal sequences (Figure 4A,B). Axial T1‐weighted sequence demonstrated relative T1 hypointensity corresponding to the abdominal mass (Figure 4C), with diffusion‐weighted sequence demonstrating restricted diffusion with low ADC value in the ADC sequence (Figure 4D). Sagittal and axial post‐contrast T1‐weighted images demonstrated heterogeneous enhancement of the corresponding abdominal mass, respectively (Figure 4E,F). Narrow our differentials, including yolk sac tumor, rhabdomyosarcoma, and lymphoma. However, imaging revealed progression of the neoplasm. The patient then underwent biopsy of the lesion, which revealed abundant eosinophilic cytoplasm, round nuclei, and variable mitotic activity (Figure 5) with immunohistochemical stain positive for SALL4, CDX2, glypican 3, and cytokeratin 7 (Figures 6, 7, 8, 9), all of which pointed towards extragonadal yolk sac tumor of the pelvis. Other recently discovered immunohistochemical markers for the tumor, like FOXA2, HNF1β, and INI1, were not stained.

## 4 Outcome and Follow‐Up

Patients' features and findings were discussed with the surgery, oncology, and gynecology teams, and were planned for the surgical removal of the mass along with laparoscopy to assess the ovarian and uterine structures. On diagnostic laparoscopy, she had a normal appearing upper abdomen, including the liver, stomach, bowels, and omentum, and there were no visible lesions or implants. The patient was then planned for surgical resection of the mass; however, after examination under anesthesia, there was concern that it would be a potentially morbid resection with a higher risk of infection, difficulty closing the area, and discussion with pediatric oncology, a decision was made to go with chemotherapy only. The patient is currently under chemotherapy and is doing well.

## 5 Discussion

Germ cell tumor consists of neoplasms with cells originating from the gonads, either the testis or the ovaries, with the yolk sac tumor being the second most common malignant ovarian germ cell neoplasm after dysgerminoma. Even though primary yolk sac tumor of the ovary is the second most malignant ovarian germ neoplasm, extragonadal yolk sac tumor in the pelvis is extremely rare, and its presentation as a subcutaneous lump is novel. Mostly, extragonadal germ cell tumors in neonates and the prepubertal age group are located in the sacrococcygeal area, while the common site of their location for females under 3 years of age is the vagina, and the mediastinum is a common location of pelvic yolk sac tumors in postpubertal females. Some of the extremely rare sites of the extragonadal germ cell tumor include the pelvis, retroperitoneum, prostate, pericardium, diaphragm, ear, eyes, penile shaft, and omentum [4].

An extra gonadal germ cell tumor has a dual mode of origin. The first origin is the remnants of primordial germ cells that become trapped outside the gonads during embryonic development, while the second origin is the transformation of somatic tumors into germ cell tumors. Extragonadal yolk sac tumor has been classified according to its demographic categories and includes congenital/neonatal, tumor in prepubertal children, and tumor in postpubertal age groups. Congenital variants of the extragonadal yolk sac tumor are often admixed with teratoma, but the tumors in the prepubertal and postpubertal age groups are pure yolk sac tumors [5].

Clinical features depend upon the specific location of the tumor and its involvement of the surrounding structures. Ovarian yolk sac tumor usually presents with features of abdominal distension, sensation of fullness, and pain, but the clinical features pertaining to extragonadal yolk sac tumor have not been described much in the literature, and the presentation as a subcutaneous lump is exceedingly rare. Prognosis depends upon age, such that the greater the age of the presentation, the worse the prognosis [6]. Diagnosis of pelvic yolk sac tumor is quite challenging and is made with history, physical examination, and various imaging modalities such as USG, CT scan, and MRI. As a radiologist and a clinician, it is often considered among the last differential diagnoses for a patient with a subcutaneous lump, and this article highlights the same rarity, focusing on its rare site and atypical presentation.

Imaging modalities play a vital role in narrowing down the differential diagnosis to an extragonadal yolk sac tumor. Three variants of patterns for a yolk sac tumor can be found in USG and usually include cystic, cystic‐solid mixed, and solid types. Cystic yolk sac tumors usually have a large cyst with regular morphology and clear boundaries. The cyst is often filled with weak echoes, septations, and a mural papillary nodule. In a cystic solid mixed tumor, the solid component has a low echo and cysts of various sizes, while in a pure solid variant, moderate echogenicity, irregular morphology, and rich blood flow can be seen in a color Doppler flow study [7]. CT demonstrates mixed hypodense and hyperdense areas corresponding to cystic and solid areas, respectively. Contrast‐enhanced CT demonstrates a solid area enhancing avidly with no enhancement of the cystic and necrotic areas. T1‐weighted image in MRI usually reveals iso to hypointense mass, and sometimes it may demonstrate foci of hyperintensity corresponding to hemorrhage, fat, or proteinaceous material, whereas the T2‐weighted sequence demonstrates hyperintensity. A heterogeneous signal can be seen in T2 T2‐weighted sequence corresponding to the areas of hemorrhage. Post‐contrast sequence demonstrates strong contrast enhancement in the solid component with no contrast enhancement for the cystic and necrotic area. DWI scan demonstrates restriction due to tightly packed cells, favoring malignancy with a corresponding low ADC value. Mass effect on adjacent pelvic structures and sometimes invasion can be noted, depending upon the grade of the tumor.

Tumor markers such as AFP are significantly elevated in patients with yolk sac tumor and serve as a marker for the detection of the tumor, monitoring the treatment, and identifying the recurrence [1]. As our patients, individuals with pelvic yolk sac tumors can often have concurrent elevated levels of CA‐125. We reviewed literature after the Year 2000 and encountered seven cases of the pelvic yolk sac tumor in the post‐pubertal age group, which are summarized in Table 1. The age of the patients ranged from 22 to 36 years, with a mean age of presentation of 29 years. Abdominal pain and fullness were common symptoms in all of them, with the AFP ranging from 1136 ng/mL to 44,1611 ng/mL and size ranging from 3 to 15.4 cm (mean, 8.3 cm). The imaging findings were characterized by hyperechoic areas on ultrasound, solid mass lesions on CT, and T2 hyperintensities on MRI. A few of the patients had necrotic areas on the mass identified by hypoechoic areas on USG and heterogeneous signal intensity in T2‐weighted imaging. Involvement of the rectum was present in two of the cases, and metastasis was present in two individuals, with one in the infracolic omentum and the other in the liver. In the majority of cases, patients underwent surgical resection of the mass followed by adjuvant chemotherapy, resulting in a tumor‐free status within a few months of completing treatment.

**TABLE 1:** Imaging findings, serum AFP level, and treatment of the pelvic yolk sac tumor in postpubertal patients.

Years	Author	Age	Imaging findings	Size	Location	AFP level	Surgery	Postop treatment	Outcome and follow up
2003	Dede et al. [8].	33	USG: hyperechoic mass CT: soft tissue mass mimicking uterine myoma	11*8*6 cm	Pelvis	1136 ng/ml	Left pelvic mass extirpation, bilateral ovarian wedge resection, and reconstruction. Pelvic, para‐aortic lymph node dissection and total omentectomy	BEP	NED, 6 months
2007	Tseng and Jung [9].	27	USG shows necrotic solid mass attached to the rectum and hemoperitoneum	6.3*6 cm	Pelvis	4830 ng/mL	Resection of the mass	EP	NED, 11 months
2008	Pasternack et al. [1].	31	CT shows semisolid, multilobulated pelvic mass separated from the normal uterus and ovaries, metastatic implant in the infracolic omentum and on hepatic capsule	7*5*3 cm	Cul de sac	8947 ng/ml	Excision of the mass and the liver nodule and omentectomy	BEP	NED, 6 months
2012	Baba et al. [10].	36	MR shows multiple disseminated lobulated tumor occupying the abdominal cavity with thickened omentum and massive ascites	Adult head size	Beneath the peritoneum occupying the whole abdominopelvic cavity	441,611 ng/ml	RSO, cytoreductive surgery	BEP	NED, 1 year
2016	Rudaitis et al. [11].	22	USG shows nonhomogeneous tumor of the pelvis CT shows Non homogenous pelvic mass, displacing the uterus and bladder, deforming and possibly infiltrating the front wall of the rectum	11.3*10.5*15.4 cm	Pelvis	13, 675 KU/l	Biopsies from a bulky tumor, peritoneal lesion, and liver metastasis	BEP	NED, 2 years
2019	Kim et al. [12].	26	USG shows well‐defined solid mass with a small central anechoic component CT shows solid mass with heterogeneous enhancement in the rectouterine pouch MRI shows solid mass with T2 intermediate to high signal intensity and internal high signal intensity component on the T1‐weighted image	9.3*7.0 cm	Pelvis	7500 ng/mL	RSO, omentectomy, and appendectomy	BEP	NED, 2 months

Abbreviations: BEP, bleomycin, etoposide, and cisplatin; EP, etoposide and cisplatin RSO, right salpingo‐oophorectomy, NED, no evidence of disease.

Predominant histopathological features include solid, tubular, and focal papillary patterns with Schiller‐Duval bodies and sinusoidal structures with fibrovascular cores lined by tumor cells. There are frequent mitotic figures with positivity for cytokeratin [13]. Yolk sac tumor demonstrates positivity with immunohistochemical markers such as alpha‐fetoprotein (AFP), glypican‐3 (GPC3), SALL4, HNF1β, and FOXA2. FOXA2 has been recognized as a master regulator for tumor formation in both yolk sac tumors of germ cell origin and somatic carcinomas with yolk sac tumor/enteroblastic differentiation (yolk sac‐like tumor). There is significant overlap of somatically derived yolk sac‐like tumor and yolk sac tumor of germ cell origin beyond morphology, including expression of the master regulators of the yolk sac tumor phenotype (FOXA2) and, in some cases, miR‐371a‐3p. However, these somatically originated yolk sac‐like tumors lack a characteristic feature of yolk sac tumors of germ cell origin, called the 12p isochromosome (i[12p]), and show significant biological and clinical differences with yolk sac tumors of germ cell origin [14]. One of the rare subtypes of yolk sac tumor is called sarcomatoid yolk sac tumor postpubertal‐type, which occurs mostly after chemotherapy. It is characterized by the downregulation of both FOXA2 and HNF1β [15]. Recently, it has also been recognized that loss of INI1 is associated with neoplasms that morphologically resemble yolk sac tumors at extragonadal sites and have both prognostic and therapeutic implications [16].

Historically, yolk sac tumor had a poor prognosis, but with the advent of adjuvant and neoadjuvant chemotherapy, the overall survival rate has increased. Common chemotherapeutic drugs used in its management include vincristine, actinomycin D, cyclophosphamide, vinblastine, bleomycin, and cisplatin. Recently, BEP therapy, including bleomycin, etoposide, and cisplatin, has been introduced for the treatment of the extragonadal yolk sac tumor with good survival results [8].

## 6 Conclusion

One of the rarest sites for extragonadal yolk sac tumor is the pelvis, with unique clinical features in each individual, depending upon the age and involvement of surrounding organs. It arises because of the failure of cellular migration with entrapment in the pelvis with subsequent tumor formation. Herein, we report a 19‐year‐old postpubertal female with complaints of a subcutaneous lump, pain, and how, with the help of the radiological investigation, we narrowed down the differential diagnosis for a rare entity in this age group. Early diagnosis of the postpubertal extra‐gonadal yolk sac tumor is essential, considering the reproductive age group and aggressive nature of the neoplasm. Only a handful of cases have been described in the literature explaining the pelvic yolk sac tumor with its clinical features and radiological findings. This case report highlights the most common imaging pictures and various modalities utilized in narrowing down the diagnosis, with the aim of keeping pelvic yolk sac tumor as an important differential while managing a case of a subcutaneous lump.

## Author Contributions

**Saurav Jha:** conceptualization, writing – original draft, writing – review and editing. **Emilee Phillips:** conceptualization, supervision. **Martin Jumbelic Safran:** writing – review and editing. **Saurabh Gupta:** supervision, writing – review and editing.

## Consent

Written informed consent was obtained from the patient to publish this report in accordance with the journal's patient consent policy.

## Conflicts of Interest

The authors declare no conflicts of interest.

## Data Availability

Data will be provided by the corresponding author upon reasonable request. Images uploaded in the separate files.
